# Synthesis of Porous Proton Ion Conducting Solid Polymer Blend Electrolytes Based on PVA: CS Polymers: Structural, Morphological and Electrochemical Properties

**DOI:** 10.3390/ma13214890

**Published:** 2020-10-30

**Authors:** Muaffaq M. Nofal, Shujahadeen B. Aziz, Jihad M. Hadi, Rebar T. Abdulwahid, Elham M. A. Dannoun, Ayub Shahab Marif, Shakhawan Al-Zangana, Qayyum Zafar, M. A. Brza, M. F. Z. Kadir

**Affiliations:** 1 Department of Mathematics and General Sciences, Prince Sultan University, P.O. Box 66833, Riyadh 11586, Saudi Arabia; muaffaqnofal@gmail.com; 2 Hameed Majid Advanced Polymeric Materials Research Laboratory, Department of Physics, College of Science, University of Sulaimani, Kurdistan Regional Government, Qlyasan Street, Sulaimani 46001, Iraq; rebar.abdulwahid@univsul.edu.iq (R.T.A.); ayub.shahab@gmail.com (A.S.M.); 3 Department of Civil Engineering, College of Engineering, Komar University of Science and Technology, Kurdistan Regional Government, Sulaimani 46001, Iraq; 4 Department of Medical Laboratory of Science, College of Health Sciences, University of Human Development, Kurdistan Regional Government, Sulaimani 46001, Iraq; jihad.chemist@gmail.com; 5 Department of Physics, College of Education, University of Sulaimani, Old Campus, Kurdistan Regional Government, Sulaimani 46001, Iraq; 6 Associate Director of General Science Department, Woman Campus, Prince Sultan University, P.O. Box 66833, Riyadh 11586, Saudi Arabia; elhamdannoun1977@gmail.com; 7 Department of Physics, College of Education, University of Garmian, Kalar 46021, Iraq; shakhawan.al-zangana@garmian.edu.krd; 8 Department of Physics, School of Science, University of Management and Technology, Lahore 54000, Pakistan; qayyumzafar@gmail.com; 9 Department of Manufacturing and Materials Engineering, Faculty of Engineering, International Islamic University of Malaysia, Kuala Lumpur 53100, Malaysia; mohamad.brza@gmail.com; 10 Centre for Foundation Studies in Science, University of Malaya, Kuala Lumpur 50603, Malaysia; mfzkadir@um.edu.my

**Keywords:** porous polymer electrolyte, XRD and morphology, impedance, electrical properties, TNM, LSV

## Abstract

In this study, porous cationic hydrogen (H^+^) conducting polymer blend electrolytes with an amorphous structure were prepared using a casting technique. Poly(vinyl alcohol) (PVA), chitosan (CS), and NH_4_SCN were used as raw materials. The peak broadening and drop in intensity of the X-ray diffraction (XRD) pattern of the electrolyte systems established the growth of the amorphous phase. The porous structure is associated with the amorphous nature, which was visualized through the field-emission scanning electron microscope (FESEM) images. The enhancement of DC ionic conductivity with increasing salt content was observed up to 40 wt.% of the added salt. The dielectric and electric modulus results were helpful in understanding the ionic conductivity behavior. The transfer number measurement (TNM) technique was used to determine the ion (*t_ion_*) and electron (*t_elec_*) transference numbers. The high electrochemical stability up to 2.25 V was recorded using the linear sweep voltammetry (LSV) technique.

## 1. Introduction

Solid polymer electrolytes (SPEs) are a rapidly growing area in the field of polymer physics. As the most significant type of polymer electrolytes (PEs), solid polymer electrolytes (SPEs) have gained the interest of researchers globally [1]. Compared to liquid or gel-based PEs, SPEs are generally more desirable due to their characteristics in terms of processability, ionic conductivity, flexibility, light weight, low cost, safety, and durability [2]. These characteristics make SPEs suitable candidates to be employed in a variety of energy storage devices such as computing laptops, cellular phones, electrochromic display devices, fuel cells, sensors, and smart credit cards [3,4]. Nowadays, the host polymers most commonly used by the researchers in the PEs are poly(vinyl alcohol) (PVA) [1,2,3,4,5,6,7], chitosan (CS) [8,9,10,11,12], methylcellulose (MC) [13,14,15], polyethylene oxide (PEO) [16,17,18], phosphomolybdic acid (PMA) [19], Nafion membranes [20], and sulfonated hydrocarbon [21].

Critical environmental issues including global warming and environmental pollution are mainly linked to human activities, particularly energy production and usage. In this context, researchers have developed new technologies to employ biodegradable polymer membranes in energy devices like dye-sensitized solar cells, fuel cells, and supercapacitors to reduce fossil fuel consumption and promote alternative energy sources [10]. Electron-donating atoms exist in the structures of polymers with functional groups, for instance, CS and PVA. The presence of lone pair electrons in the oxygen or nitrogen atoms is essential for cation and anion dissociation of the added salt, meaning that the functional groups are important complexation sites for coordination of cations [8,10]. Different approaches including polymer blending and using various salts were investigated to overcome the poor conduction of PE systems. As mentioned in the literature, inorganic salts, particularly alkali metal salts, can significantly improve the conductivity of SPEs and make them reliable for many energy device applications, such as lithium-ion batteries and dual-ion capacitors [22,23,24,25,26]. PVA is a semicrystalline polymer with many hydroxyl functional groups attached to its backbone structure. This polymer is widely used because of its interesting properties like biodegradability, nontoxicity, low cost, biocompatibility, high charge storage capacitance, and high stability [27,28]. On the other hand, chitosan (CS) is a well-known biopolymer in nature obtained from partial deacetylation of chitin. Unlike PVA, CS is a natural semicrystalline polymer with a structure containing two reactive polar groups, amino (NH_2_) and hydroxyl (OH), which are suitable for modification [29]. Furthermore, the biomedical applications of CS due to its special characteristics, such as facile processability, biodegradability, antibacterial properties, and cost-effectiveness, highlight the eco-benign properties of this natural polymer [30,31]. CS has several promising pharmaceutical uses and has been studied as a new carrier material in drug delivery systems and for its wound healing, fat binding, antibacterial, and hypocholesterolemic effects [32]. Biopolymer-based membranes, for example, CS, carrageenan, and cellulose, are considered as some good alternative materials to improve the performance of fuel cells, since they are eco-friendly and cheap [33]. CS is one of the most suitable materials that can be used as a substitute for the Nafion membrane since it has shown enhanced performance in low-temperature fuel cell applications [34,35]. In addition, CS exhibited good proton conductivity, attractive alcohol barrier property, and thermal stability after it goes through the crosslinking mechanism [34,35].

Mixing two or more polymer materials (known as the polymer blend approach) has been found to be a suitable technique to reduce the crystallinity degree and increase ionic conductivity at room temperature. Kadir et al. reported the potential use of PVA and CS polymers for applications in electrochemical devices [28,36]. In the polymer blend, electrolytes (usually metal salts) are used as ionic conductivity enhancers. Proton-conducting PEs have negatively charged groups attached to the polymer backbone, and the charge carriers in these systems are found to be H^+^ ions, which can be obtained from the dissociation of various ammonium salts [36]. During the dissociation process of ammonium salts, the NH_4_^+^ ions with other anions are obtained [37]. Three hydrogen atoms in the NH_4_^+^ ion are tightly bonded, whereas the fourth one is weakly bonded and can hop in the polymer host matrix under the influence of an applied electric field [37]. An extensive survey of the literature illustrates that some ammonium salts, such as ammonium nitrate (NH_4_NO_3_), ammonium chloride (NH_4_Cl), ammonium bromide (NH_4_Br), ammonium iodide (NH_4_I), and ammonium thiocyanate (NH_4_SCN), have been used as dopants since they are good proton (H^+^) providers [2,13,14,15,27,28,36]. It is crucial to note that some of the ammonium salts, including NH_4_SCN, can have hazardous properties, and potential toxicity should be considered during experimental work.

The intensive survey of the literature revealed that the structural and electrochemical properties of PVA/CS/NH_4_SCN electrolytes have not been investigated yet. Therefore, in this work, a proton-conducting PE was prepared using low-lattice-energy NH_4_SCN salt [38] as a proton provider. In our previous work, we comprehensively showed that it is crucial to consider the lattice energy of salts in the preparation of polymer electrolytes [39]. In the current study, PVA/CS blended system was doped with various amounts of NH_4_SCN salt. The structural, morphological, and electrochemical properties of the blended PEs based on PVA/CS were also been investigated in order to understand the structure–property relationships.

## 2. Experimental

### 2.1. Material and Preparation of Blended SPE Films

In this work, the polymer blend was prepared from the chitosan (CS) and poly(vinyl alcohol) (PVA). Ammonium thiocyanate (NH_4_SCN) salt (purity 99%) and acetic acid (1%) (CH_3_COOH) were used as doping salt and solvent, respectively. All chemicals were used as obtained from Sigma-Aldrich without further purification. The solution casting technique was employed to prepare PVA/CS/NH_4_SCN blend SPE films from the raw materials. First, 10 mL of acetic acid was added into 990 mL of distilled water. To prepare the electrolyte samples, 0.5 g of CS was dissolved in 30 mL of 1% acetic acid and left for 3 h under stirring with a magnetic stirrer at room temperature. Simultaneously, 0.5 g of PVA was dissolved completely in 20 mL of distilled water for 1 h under stirring with a magnetic stirrer at 80 °C and then allowed to cool down to room temperature. Later, both PVA and CS solutions were mixed in a beaker with a magnetic stirrer through continuous stirring for 4 h to obtain a homogeneous solution. Various amounts of NH_4_SCN dopant salt were added to the CS/PVA solutions, separately in different beakers, while stirring continuously for 6 h until clear solutions were obtained. Five samples with different weight percentages of salts, ranging from 10 to 50 wt.% in 10 wt.% steps, were prepared and coded as PCSH1, PCSH2, PCSH3, PCSH4, and PCSH5, respectively. Finally, the mixtures were transferred into uncovered Petri dishes, allowing the solvent to evaporate gradually at ambient temperature for 3 weeks to obtain completely dry SPE films. The films were dried at room temperature to provide solvent-free films and stored in a desiccator with blue silica gel to remove moisture. The thickness of the samples was measured and found to be in the range of 119 to 123 µm. Table 1 summarizes the weight ratios of samples.

### 2.2. XRD and FESEM Study

A Siemens D-5000 X-ray diffractometer (Bruker AXS GmbH, Berlin, Germany) at the wavelength (λ) of 1.5406 A° was employed to record the XRD pattern of PVA/CS/xNH_4_SCN with operating current and voltage of 40 mA and 40 kV, respectively. The samples were scanned in the step-scan mode with 2θ angle ranging from 10 to 80° with a step size of 0.1°.

The surface morphology and structural properties of the prepared polymer blend electrolyte films were investigated by field-emission scanning electron microscopy (FESEM) (FEI Quanta 200 FESEM) (FEI Company, Hillsboro, OR, USA).

### 2.3. Electrical Impedance Spectroscopy EIS

The study of electrical impedance behavior of the fabricated blend films was performed using LCR (HIOKI 3531 Z Hi-tester, Nagano, Japan) impedance analyzer in the frequency range of 50 Hz to 5 MHz, which was interfaced to a computer for data acquisition. For this reason, a couple of stainless-steel (SS) electrodes 2 cm in diameter were used as blocking electrodes. These electrodes are provided with special springs to squeeze and maintain the sandwiched film in an optimal pressure. Then, the films were cut into small circles with a diameter of 1.6 cm and sandwiched between the electrodes for dielectric measurements. The Nyquist plots for complex impedance (Z*) real part *Z’* and imaginary part *Z”* were obtained; from these, the bulk resistance (*R_b_*) for each sample was determined by the intercept with the real axis. Both real and imaginary parts of complex permittivity (ε^*^) and complex electric modulus (M*) were also calculated from the impedance data (i.e., *Z’* and *Z”*).

### 2.4. Transfer Number Measurement (TNM)

The V&A instrument DP3003 was employed for measuring the PCSH4 and PCSH5 blend samples through DC polarization technique versus time at room temperature. TNM for each sample was calculated by tracking a digital DC to V&A instrument DP3003 (V & A Instrument, Shanghai, China), while the cell was polarized with an applied voltage of 0.2 V.

### 2.5. Linear Sweep Voltammetry LSV

The principal goal of utilizing LSV was to evaluate the electrochemical stability of the most conductive film, assess the working potential range, and determine the breakdown voltage at room temperature. This technique was conducted using a Digi-IVY DY2300 potentiostat (Neware, Shenzhen, China) at the scan rate of 10 mV s^−1^ in the voltage potential range of 0 to 3 V. The films were kept between the SS electrodes in order to keep the sample at ambient temperature.

## 3. Result and Discussions

### 3.1. XRD Analysis

**Figure 1:**
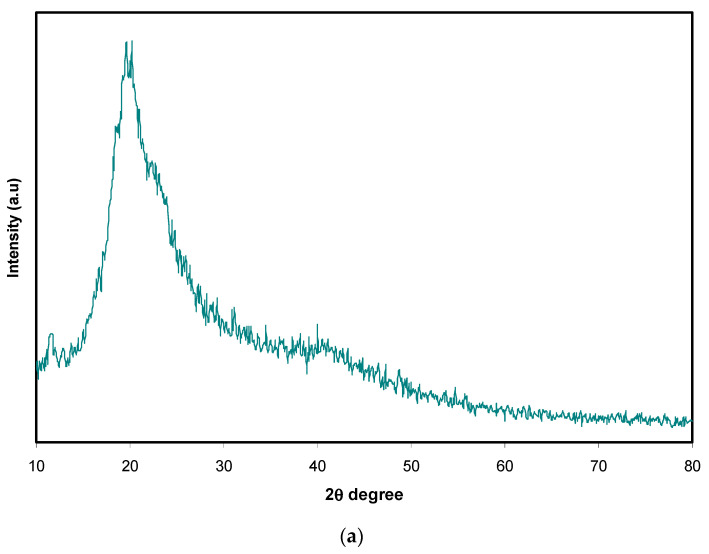

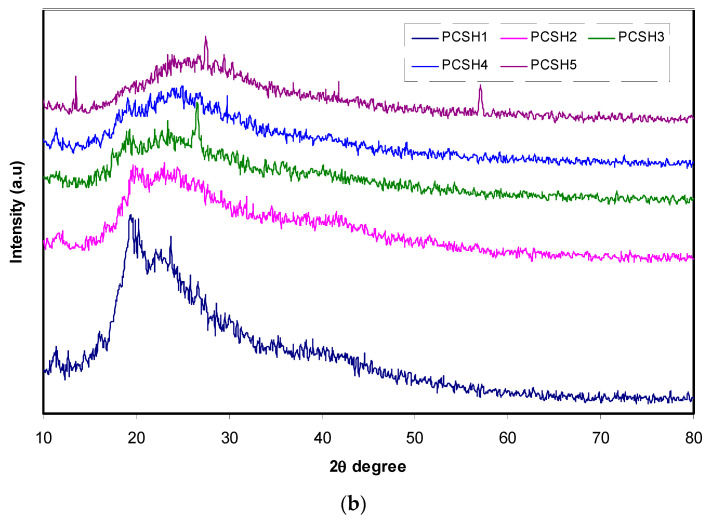
X-ray diffraction (XRD) spectra for (**a**) pure PVA/CS polymer blend and (**b**) PVA/CS/NH_4_SCN electrolyte systems at room temperature.

X-ray diffraction is a technique that is widely used to identify amorphous and the crystalline phases of electrolyte film samples. The XRD pattern was used to examine the structural changes of the selected SPE film blends. Figure 1a,b illustrates the X-ray diffraction pattern of pure PVA/CS and doped blend systems at room temperature [15,40]. Both pure PVA and CS exhibit semicrystalline behavior [27]. However, through the blending of these two polymers, the peaks became broader, with a drop in their intensities indicating the increase in the amorphous region in the blended system (as shown in Figure 1a). It can be noticed that the increase of the NH_4_SCN salt concentration up to 40 wt.% suppressed the intensity of the peaks of the PVA/CS blend film. This implies that a clear interaction occurred between the dopant salt and the polymer blend, which resulted in the disappearance of the sharp crystalline peaks of the NH_4_SCN salt. Further addition of the salt, reaching 50 wt.%, resulted in the appearance of some sharp peaks, which could be related to the occurrence of salt recrystallization. This is related to the salt recrystallization process, which occurred at high NH4SCN content. For example, a crystalline peak at about 2θ=27° appeared in PCSH3 and PCSH4 and shifted to 2θ≈28° in PCSH5; this peak did not exist in other samples. Similarly, another crystalline peak appeared at around 2θ=58° for the PCSH5 sample and did not exist in the rest of the samples. These peaks are clear evidence of the recrystallization process at high salt content. These observations are also supported by the results obtained using the FESEM technique showing that the surface roughness of the PCSH5 electrolyte film increased (as discussed in a later section). This is referring to the ion’s recombination and formation of ion clusters at the film surface, which caused the loss of a large number of ionic species and the reduction of conductivity [36,41]. Previous studies suggested that the amorphous nature, which is more favorable for the conduction in the Pes, is highlighted by the widening of the X-ray diffraction peaks, also suggesting that the DC ionic conductivity is greater for larger amorphous regions. The variation in the free volume of a polymer influences its molecular motion and physical properties. The polymer blends based on PVA/CS lead to additional free volume, which in turn speeds up the migration of ions at an optimal concentration of the dopant salt. It can be detected that the development of the amorphous region with increasing concentration of the NH_4_SCN salt is due to the higher intra- and intermolecular forces of hydrogen bonding, which results in the polymer chain structure having softer nature [42,43]. Thus, the broader peak in the XRD pattern of PCSH4 film electrolyte is considered indicative of some kind of interaction between the blended polar polymer and the dopant salt. The PCSH4 electrolyte had the maximum DC ionic conductivity, as will be discussed later. Finally, the inorganic salt of NH_4_SCN has shown a great influence on reducing the crystallinity phases of PVA/CS blended SPEs, since both PVA and CS are semicrystalline polar polymers [8,10].

### 3.2. Morphology Study

**Figure 2:**
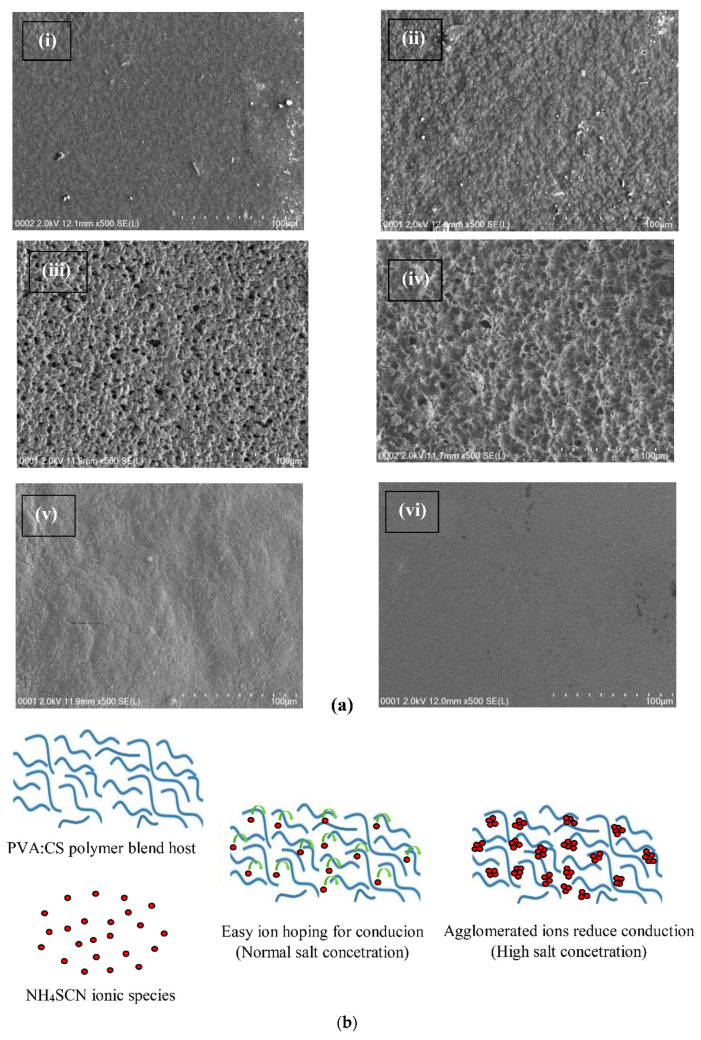
(**a**) FESEM micrographs of (i) PCSH1, (ii) PCSH2, (iii) PCSH3, (iv) PCSH4, (v) PCSH5, and (vi) pure PVA/CS blend electrolytes. (**b**) Schematic illustration of ionic transfer in normal NH_4_SCN salt concentration and blockage of pathways caused by ion agglomeration in high NH_4_SCN salt concentration.

Field-emission scanning electron microscopy (FESEM) is a convenient technique for inspecting the surface composition and morphology of numerous SPE systems [44]. Figure 2a (i–vi) presents the FESEM micrograph images of pure and PVA/CS blends doped with different concentrations of NH_4_SCN salt. The FESEM image of pure PVA/CS (Figure 2a (vi)) looks smooth and uniform, which is a good sign of interaction between the blended polymers. It is obvious that the FESEM image of the PCSH1 electrolyte sample shows no porous formation with no phase separation, and the prepared sample appeared to be homogeneous. For the PCSH2 sample, the presence of some white particles on the surface of the film can be observed, which are related to the formation of salt particles without being involved in the ionic conduction process. Moreover, these white particles reduce the ion concentrations that play a vital role in ion-conduction-based electrolytes [41,45]. In fact, as the salt content increased, the surface morphology of the films was changed substantially. Nonetheless, a porous homogenous structure without any protruded particles can be seen for both PCSH3 and PCSH4 samples, which provides more space inside the films with a high surface area. Correspondingly, the more spacious area provides a larger pathway for charge transportation, which is important for ionic conductivity in the PEs [46]. On the other hand, for the highest salt content (50 wt.%), the surface roughness of the electrolyte film increased and its porosity drastically decreased due to the agglomeration of ions, as illustrated schematically in Figure 2b. This can be demonstrated by the fact that the aggregated ions can protrude through the surface of the film. This phenomenon is shown in the XRD pattern where the amorphous region is enlarged as the concentration of the NH_4_SCN salt is increased up to 40 wt.% [45,47].

### 3.3. Impedance Analysis

**Figure 3:**
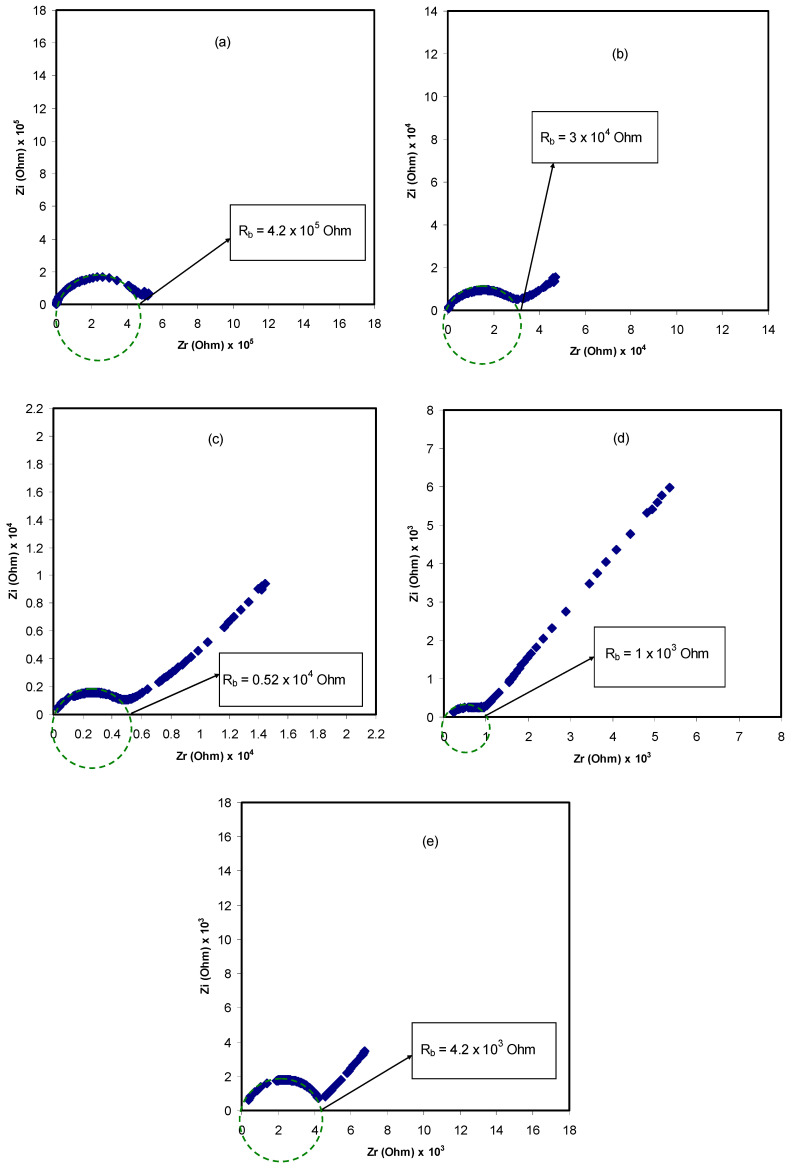
Impedance plot (*Z_i_* versus *Z_r_*) of blended solid polymer electrolytes at room temperature for (**a**) PCSH1, (**b**) PCSH2, (**c**) PCSH3, (**d**) PCSH4, and (**e**) PCSH5.

Electrical impedance spectroscopy (EIS) is considered an effective method for explaining the transference of ions at the sample/electrode interface region and the electrical properties of the electrolyte material. AC impedance analysis can be used to determine the ionic conductivity of the electrolyte films, which mainly depends on the mobility and the density of the charge carriers in the electrolyte films [48,49]. Figure 3a–e illustrates the impedance spectra of the Nyquist plots (*Z_i_* versus *Z_r_*) for all the blended SPE samples at room temperature. Through this technique, the effect of the NH_4_SCN dopant salt concentration on the ionic conductivity of the PVA/CS blended samples was explored. It is clear from the Nyquist plots that the half-circle diameter is reduced with the increase of NH_4_SCN salt content for the set of the samples up to 40 wt.%. However, upon further addition of NH_4_SCN salt up to 50 wt.%, the diameter of the semicircle is increased. This is caused by the agglomeration of ions in the polymer–salt complexes in the system at the highest added salt concentration [14,50,51]. This in turn could reduce the percentage of free space of the charge carriers and lead to a drop in the ionic conductivity of the system. The bulk resistance (R_b_) values for all the blended SPE systems were obtained by the intersection of the real axis (Zr) with the spike line. The value of *R_b_* for the PCSH1 was found to be 4.2 × 10^5^ Ohm, and this value decreased to 10^3^ Ohm for the PCSH4 electrolyte sample. Besides, the DC conductivity was calculated using the following relation [52]:

(1) $$\sigma_{dc}=\;\left[\frac{1}{R_{b}}\right]\;\times \;\left[\frac{1}{A}\right]$$

where *t* is the thickness of the electrolyte film, *A* is the area of the electrode, and *R_b_* is the bulk resistance of the material [52]. Moreover, the maximum DC ionic conductivity was found to be 1.48 × 10^−5^ S cm^−1^ for the 40 wt.% NH_4_SCN doped salt at room temperature (listed in Table 2), which indicates the highest ion mobility with the largest amorphous region among the PCSH electrolyte systems. The impedance spectroscopy outcomes are in good agreement with the FESEM and XRD results. In addition, the ionic conductivity of the system increased as the salt content reached to 40 wt.%. However, at 50 wt.% of NH_4_SCN salt, the value of *R_b_* increased and the conductivity decreased due to the recombination of cations and anions of the salt, which dropped the number of movable ions and slowed the motion of ions in the polymer blend network [53]. In polymer electrolytes containing ammonium salts, protons (H^+^) are transported by means of the Grotthuss mechanism [54]. As mentioned in Section 1, one of the hydrogen atoms is weakly bonded and can jump in the polymer matrix under the influence of an external electric field [37]. The mobility of the H^+^ ion from one site to another is a chance outcome in which the mobile H^+^ ion is replaced by another proton from an adjacent site. This is the Grotthuss mechanism where proton transfer happens through proton replacement between the polymer–salt complexed sites [54]. In comparison, the maximum DC ionic conductivity for a CS/PVA/NH_4_NO_3_ system was reported to be 2.07 × 10^−5^ S cm^−1^ by Kadir et al. [36], which is very close to the DC conductivity value found in this study. In addition, the DC conductivity for a CS/PVA/NH_4_I system was found to be 1.77 × 10^−6^ S cm^−1^ by Buraidah and Arof [27]. Shukur et al. investigated a chitosan/NH_4_Br system without blending with another polymer, and the highest obtained conductivity was 4.2 × 10^−7^ S cm^−1^ [55]. All these findings confirm that polymer blending is a proper method to boost the ionic conductivity in the polymer-based electrolytes.

### 3.4. Dielectric and Electrical Modulus Study

**Figure 4:**
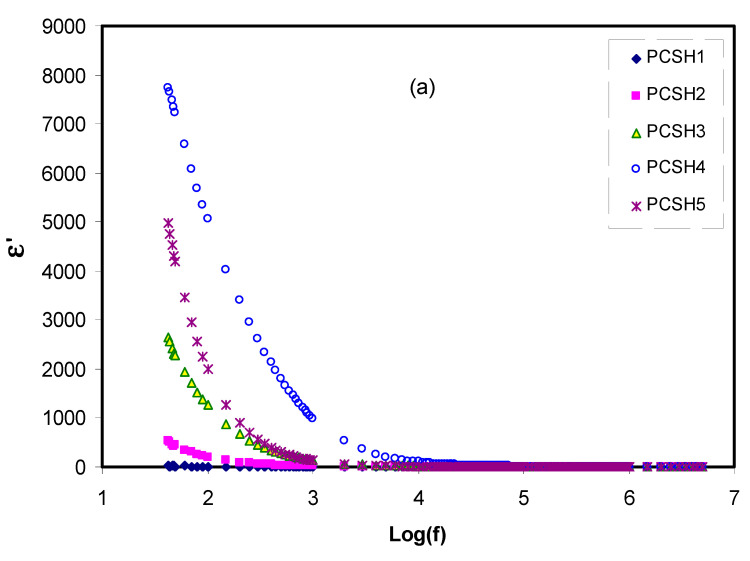

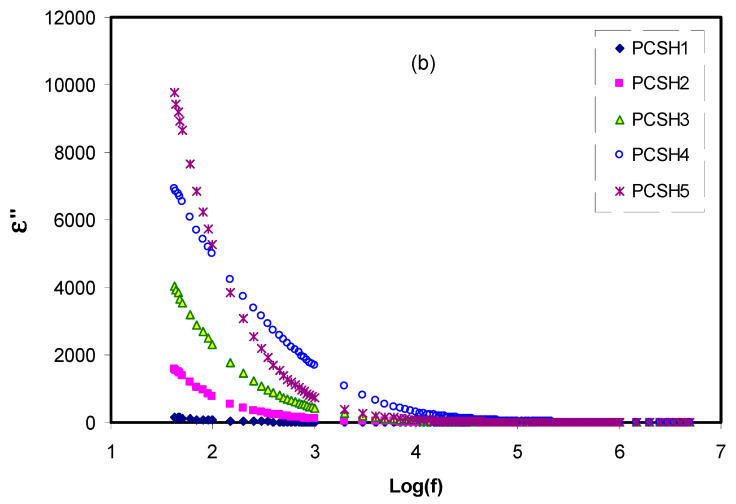
Variation of (**a**) dielectric constant (ε′) and (**b**) dielectric loss (ε″) with frequency for the polymer blend electrolyte samples.

Dielectric properties give information on the ionic transference mechanism in a polymer-based electrolyte. Through these properties, the effect of polarization and nature of the ion conduction inside the electrolyte films can be characterized [46,56]. In addition, the nature of interactions between blended polymers has been evaluated using this technique [46,56]. For the PCSH blended SPE systems, the dielectric properties were determined based on the plots of dielectric parameters (ε′, ε″). These plots are generated from the impedance data (i.e., *Z’* and *Z”*) by utilizing the following equations [56]:

(2) $$\varepsilon^{\prime }=\frac{Z^{\prime \prime }}{\omega C_{o}\;\left(Z^{\prime }{}^{2}+Z^{\prime \prime }{}^{2}\right)}$$

(3) $$\varepsilon^{\prime \prime }=\;\frac{Z^{\prime }}{\omega C_{o}\;\left(Z^{\prime }{}^{2}+Z^{\prime \prime }{}^{2}\right)}$$

where *ω* is the angular frequency of the applied filed (*ω = 2πf*); ε′ and ε″ are the dielectric constant and dielectric loss, respectively; and *C_o_* is the vacuum capacitance, which is given by *ε_o_A/t,* where *ε_o_* is the permittivity of free space, *A* is the electrode cross-sectional area, and *t* is the film thickness. Figure 4a,b presents the real part (ε′) and the imaginary part (ε″) of the dielectric parameters as a function of frequency at room temperature for the polymer blend electrolyte samples. It can be observed that the dielectric permittivity parameters (ε′, ε″) are maximum at low-frequency regions owing to electrode polarization (EP) and high density of charge carriers [56,57,58]. In contrast, at the high-frequency region (>10 kHz), the values of dielectric permittivity are significantly not dependent on frequency. A sharp rise in the dielectric constant (ε′) was particularly recorded for the PCSH4 electrolyte due to the existence of the high number of ions and maximum ionic conductivity. The PCSH1 exemplified roughly constant behavior, whereas the PCSH2 showed a steady increase. Correspondingly, the ion dispersion was reduced as the dielectric constant dropped quickly at the high-frequency region, decaying to a constant value of 1.2 when log (f) reached above 4 [59,60]. It is obvious in Figure 4a as the amount of the doped salt increased up to 40 wt.% of NH_4_SCN, the value of the dielectric constant gradually increased. However, for 50 wt.%, the values of the dielectric constant (ε′) dropped due to the aggregation of ions and the decrement of DC conductivity [44,51].

In addition, the electric modulus was determined to better understand the conductivity and bulk relaxation process in the blended SPE systems. Through the electrical modulus formalism, space charge phenomena and electrode polarization (EP) problems can be diminished, since it is the reciprocal of the dielectric complex and can be used to minimize the effect of electrode polarization at the high-frequency region. The imaginary (*M_i_*) and real parts (*M_r_*) of complex modulus (M*) of PVA/CS/xNH_4_SCN blend SPEs were determined from the impedance data (i.e., *Z’* and *Z”*) by utilizing the following relations [56]:

(4) $$M^{\prime }=\;\frac{\varepsilon^{\prime }}{\;\left(\varepsilon^{\prime }{}^{2}+\varepsilon^{\prime \prime }{}^{2}\right)}=\omega C_{o}Z^{\prime \prime }$$

(5) $$M^{\prime \prime }=\;\frac{\varepsilon^{\prime \prime }}{\;\left(\varepsilon^{\prime }{}^{2}+\varepsilon^{\prime \prime }{}^{2}\right)}=\omega C_{o}Z^{\prime }$$

**Figure 5:**
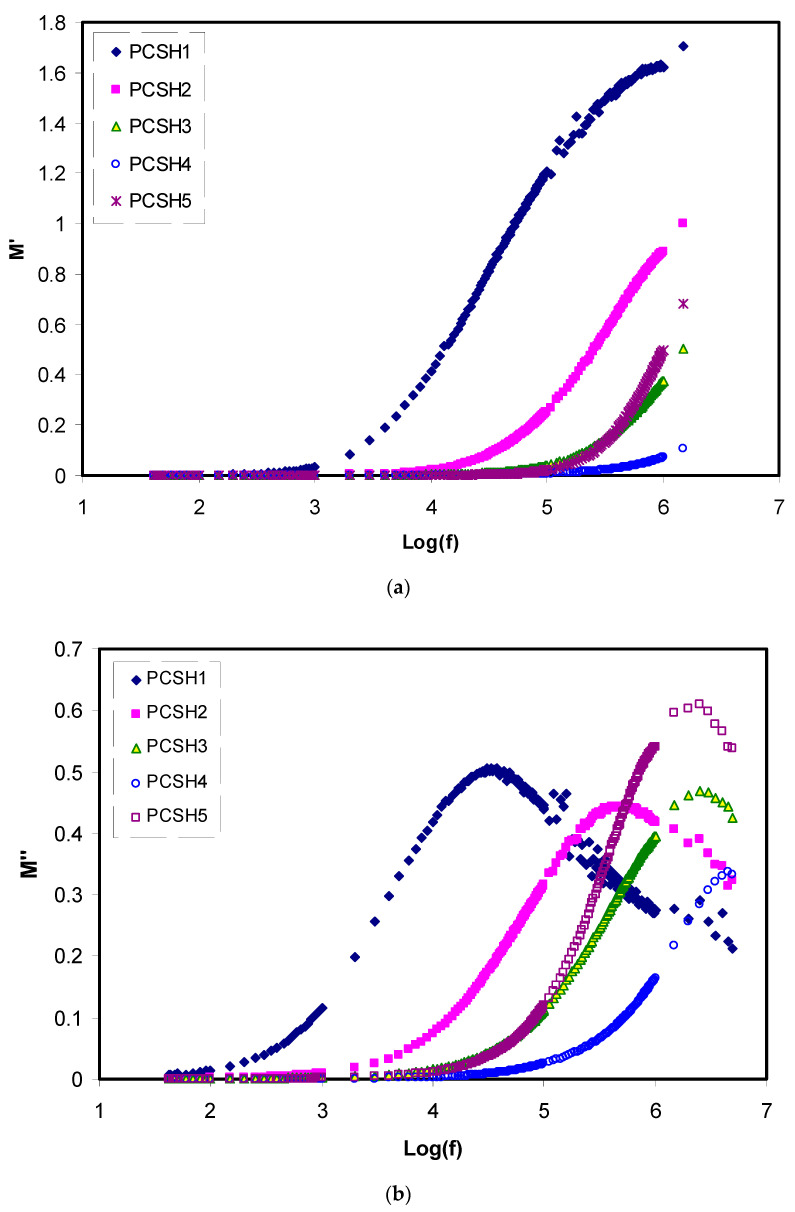
Variation of (**a**) real part of electric modulus (M_r_) and (**b**) imaginary part of electric modulus (M_i_) versus frequency for the polymer blend electrolyte systems at room temperature.

The frequency dependences of the real part (*M_r_*) and imaginary part (*M_i_*) of electric modulus are presented in Figure 5a,b for the PCSH electrolyte films versus frequency at room temperature [61,62]. It is clear that the values of both real and imaginary parts (i.e., *M_r_* and *M_i_*) are increased at the higher frequency region and reach the maximum value, which is attributed to the decrement of the EP and dielectric constant at the high-frequency region. However, the presence of the relaxation peaks in the imaginary part of electric modulus (*M_i_*) reveals that the PCSH blend electrolytes samples are ionic conductors, which means the behavior is non-Debye type. On the other hand, the low value, almost reaching zero, of both real and imaginary parts (i.e., *M_r_* and *M_i_*) can be observed in the low-frequency regions. This is due to the overthrow of the EP influence [63,64]. In the modulus plots, among the NH_4_SCN concentrations, the PCSH4 shows the minimum intensity of modulus, indicating the highest DC ionic conductivity.

### 3.5. Transfer Number Measurement (TNM) Study

**Figure 6:**
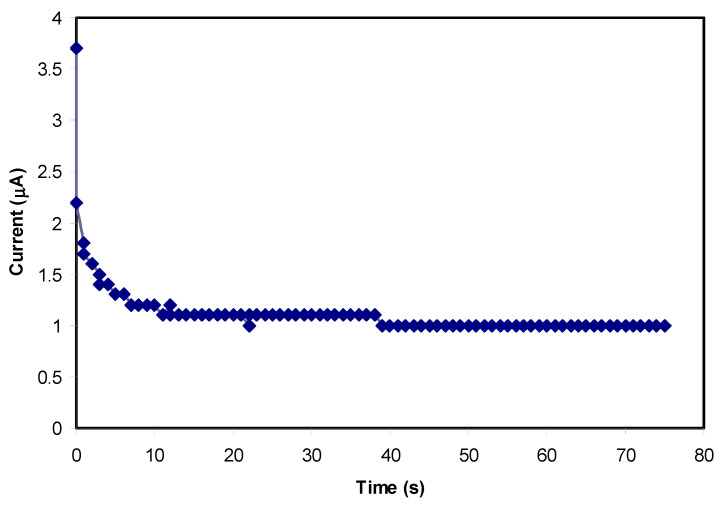
Polarization current for PCSH4 conduction sample versus time.

**Figure 7:**
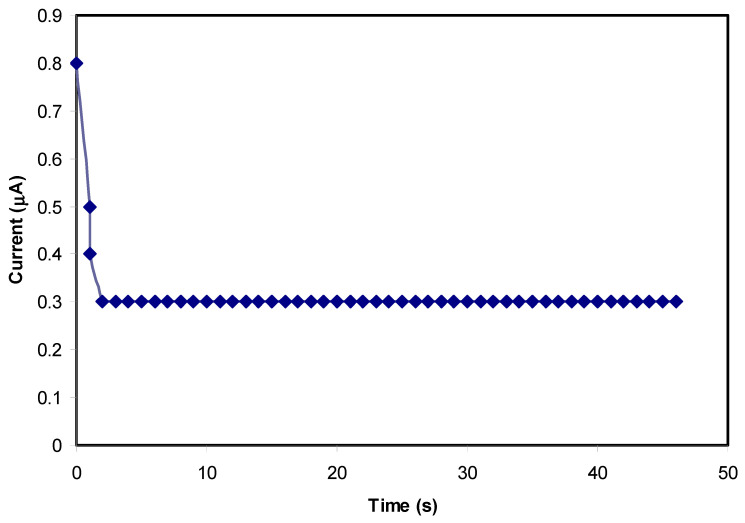
Polarization current for PCSH5 conduction sample versus time.

The TNM technique was employed successfully to estimate the behavior of the particular conduction species in the PVA/CS/NH_4_SCN blend SPE systems using the DC polarization process. Additionally, to confirm the real behavior of the ions in the PCSH blended SPE films, the TNM study is vital. Regarding this, the nature of ions can be determined by calculating the overall transference number of ions (t_ion_), which is responsible for the DC ionic conductivity in the polymer-based electrolytes [65,66]. Figure 6 and Figure 7 show the plots of polarization current against time at the applied voltage of 0.2 V for the PCSH4 and the PCSH5 films having 40 wt.% and 50 wt.% NH_4_SCN salt added, respectively. Clearly, from the TNM schemes, it can be seen that the initial total current flow dropped quickly and reduced with time. The electrolyte was polarized once it reached the steady state, whereas, the rest of the current stream was a result of electron carriers due to the blocking of cations and anions by stainless-steel (SS) electrodes, which only allow electrons to pass [67,68,69,70]. The following equations were used to determine the values of both electron (*t_e_*) and the ion (*t*_ion_) transference numbers [38]:

(6) $$t_{ion}\;=\;\frac{I_{i}-I_{ss}}{I_{i}}\;\;$$

(7) $$t_{ion}\;=\;1-\;t_{e}$$

where *I_i_* is the initial current, which is distributed to the ions and electrons, and *I_ss_* is the steady-state current that includes electrons only. The result of the ion transference (*t_ion_*) was determined from the extracted values of the initial and the steady-state currents for the PCSH4 and PCSH5 conduction samples, which were found to be 0.72 and 0.65, respectively. Therefore, the electron transference (*t_e_*) values were 0.28 and 0.35. Obviously, the results achieved from the TNM study agree with the EIS results in which the most conductive sample (PCSH4) had the maximum DC ionic conductivity [38,71].

The SCN^−^ anion of the NH_4_SCN salt has a large ionic radius of 2.5 Å [72]. Larger-sized ions have lower mobility in comparison with small-size ions as it is difficult for the former to move within the electrolyte and reach the SS electrode surface. This might be the reason for the small value of *t_ion_* in the PCSH4 system, which is not very close to the ideal value. Furthermore, the value of the DC conductivity of the most conductive film (PCSH4) is in agreement with the value of the t_ion_ from the TNM analysis.

### 3.6. Linear Sweep Voltammetry (LSV) Study

**Figure 8:**
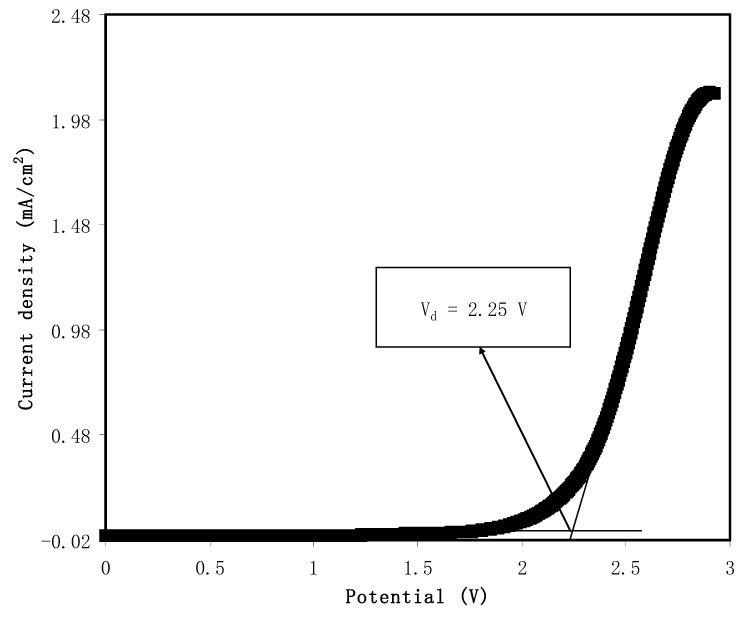
Linear sweep voltammetry plot for the PCSH4 sample at room temperature.

Another critical parameter of SPEs used in energy storage device applications is the electrochemical stability. Through employing linear sweep voltammetry (LSV), the electrochemical stability of the largest conducting sample of PVA/CS/NH_4_SCN system was investigated at 10 mV s^−1^. Figure 8 represents the LSV plot of the most conductive PCSH4 film sample at room temperature. A decomposition potential series was measured in the range of 0 to 3 V at the scan rate of 10 mV s^−1^ for the prepared cells using SS electrodes, in which no clear increase in the value of current was observed up to 2.25 V [73,74,75]. The decomposition voltage of the sample was calculated to be 2.25 V at ambient temperature. The sharp ascent of the current density was observed beyond 2.25 V, which is due to the decomposition of the sample at the surface of the SS electrodes. A high electrochemical stability window was obtained for the polymers with the presence of a strong electron-withdrawing group [76,77,78]. The minimum requirement of breakdown voltage for polymer-based electrolytes that can be used in electrochemical device applications is supposed to be ~1 V [17]. Based on the value of the decomposition potential, the PCSH4 film is suitable to be used in energy storage device applications. Table 3 reveals the decomposition voltage for several systems reported in the literature. Previous studies illustrated that in addition to high electrochemical stability, PEs must possess high DC conductivity (10^−5^ to 10^−3^ S/cm) to be used for electrochemical device applications [79,80,81]. In our future work, we will focus on the plasticization of the most conductive system of the present work to improve its DC conductivity and TNM value for energy storage application.

## 4. Conclusions

In conclusion, the effects of polymer blending technique and NH_4_SCN dopant salt on the structure, morphology, and ionic conductivity of the PVA/CS blended SPE have been intensively investigated. Both XRD and FESEM outcomes of the PCSH samples have shown that the polymer blending method allows the blended host polymer to develop a greater free volume, which in turn enhances the ionic mobility. Moreover, it has been shown that the inorganic salt of NH_4_SCN has a substantial influence on reducing the crystalline phases of PVA/CS polymer blends. The impedance spectroscopy of the samples revealed that 40 wt.% of NH_4_SCN is an optimum doping concentration value, resulting in the PCSH4 film having the highest ionic conductivity (1.48 × 10^−5^ S cm^−1^). It was shown that further increment in the salt content caused the conductivity to drop due to the salt recrystallization. Additionally, dielectric properties (ε*, M*) and TNM and LSV studies confirmed that the amorphous nature, porosity, and conductivity of the systems were enhanced by increasing the amount of NH_4_SCN up to 40 wt.%, which is favorable for electrochemical device applications. Subsequently, the PCSH4 system displayed a high electrochemical stability window of up to 2.25 V through the LSV test. The rise in the dielectric parameters (ε′, ε″) signifies that the increase in the conductivity is mainly due to the intensification of the number of ion species. The outcomes of the current study are promising and highlight that choosing the appropriate polymer blend with a suitable amount of dopant salt can make the fabricated solid polymer electrolytes suitable for electrochemical device applications.

## Figures and Tables

**Table 1:** Designation and composition of poly(vinyl alcohol) (PVA)/chitosan (CS)/NH_4_SCN blend solid polymer electrolyte (SPE) systems.

Sample Designation	(PVA) (g)	(CS) (g)	NH_4_SCN wt%
PCSH1	0.5	0.5	10
PCSH2	0.5	0.5	20
PCSH3	0.5	0.5	30
PCSH4	0.5	0.5	40
PCSH5	0.5	0.5	50

**Table 2:** The DC conductivity value of PVA/CS/xNH_4_SCN systems.

Sample Code	DC Conductivity (S/cm)
PCSH1	(3.43 × 10^−8^) ± 0.32
PCSH2	(4.84 × 10^−7^) ± 0.54
PCSH3	(2.76 × 10^−6^) ± 0.29
PCSH4	(1.36 × 10^−5^) ± 0.19
PCSH5	(3.49 × 10^−6^) ± 0.39

**Table 3:** The comparison of decomposition voltage of various SPEs.

SPEs	Scan Rate mV s^−1^	Electrode Material	Decomposition Voltage (V)	References
PVA/dextran/NH_4_I	50	Stainless steel	1.3	[82]
PEO/NH_4_SCN/CeO_2_	5	Stainless steel	1.4	[83]
PVA/CS/NH_4_Br	1	Stainless steel	1.57	[84]
PVA/CS/NH_4_NO_3_/EC	10	Stainless steel	1.7	[85]
CS/PS/NH_4_F	100	Stainless steel	1.78	[86]
CS/AgNO_3_/Al_2_O_3_/glycerol	10	Stainless steel	1.8	[87]
MC/PS/NH_4_NO_3_/glycerol	1	Stainless steel	1.88	[88]
MC/PS/LiClO_4_	5	Stainless steel	2	[89]
CS/MC/NH_4_I	10	Stainless steel	2.1	[90]
PVA/proline/NH_4_SCN	1	Stainless steel	3.61	[91]
PVA/proline/NH_4_Cl	1	Stainless steel	3.10	[92]
MC/NH_4_NO_3_/PEG	1	Stainless steel	2.4	[93]
PVA/CS/NH_4_SCN	10	Stainless steel	2.25	This work
